# User Activity Recognition in Smart Homes Using Pattern Clustering Applied to Temporal ANN Algorithm

**DOI:** 10.3390/s150511953

**Published:** 2015-05-21

**Authors:** Serge Thomas Mickala Bourobou, Younghwan Yoo

**Affiliations:** Department of Electrical and Computer Engineering, Pusan National University, Busandaehak-ro 63beon-gil, Geumjeong-gu, Busan 609-735, Korea; E-Mail: thomaserge@yahoo.fr

**Keywords:** activity recognition, Allen’s temporal relations, anomaly prediction, neural network, pattern clustering, smart home

## Abstract

This paper discusses the possibility of recognizing and predicting user activities in the IoT (Internet of Things) based smart environment. The activity recognition is usually done through two steps: activity pattern clustering and activity type decision. Although many related works have been suggested, they had some limited performance because they focused only on one part between the two steps. This paper tries to find the best combination of a pattern clustering method and an activity decision algorithm among various existing works. For the first step, in order to classify so varied and complex user activities, we use a relevant and efficient unsupervised learning method called the K-pattern clustering algorithm. In the second step, the training of smart environment for recognizing and predicting user activities inside his/her personal space is done by utilizing the artificial neural network based on the Allen’s temporal relations. The experimental results show that our combined method provides the higher recognition accuracy for various activities, as compared with other data mining classification algorithms. Furthermore, it is more appropriate for a dynamic environment like an IoT based smart home.

## 1. Introduction

The Internet of Thing (IoT) is the interaction of ubiquitous everyday sensors and devices to link physical and virtual objects through seamless networks. To construct a novel paradigm “anytime, anywhere, any service for anyone”, the IoT involves various heterogeneous techniques. The opportunities offered by the IoT make it possible to provide various applications based on it. Among them, the smart home is a sophisticated research field in smart automation systems of which the overall motive is the enhancement of users’ comfort and the guarantee of their safety and security conditions with minimal operation costs. Since a smart home is an automated environment, it has the capability to monitor, detect and record daily activity patterns by using different types of sensors and communication technologies.

**Figure 1:**
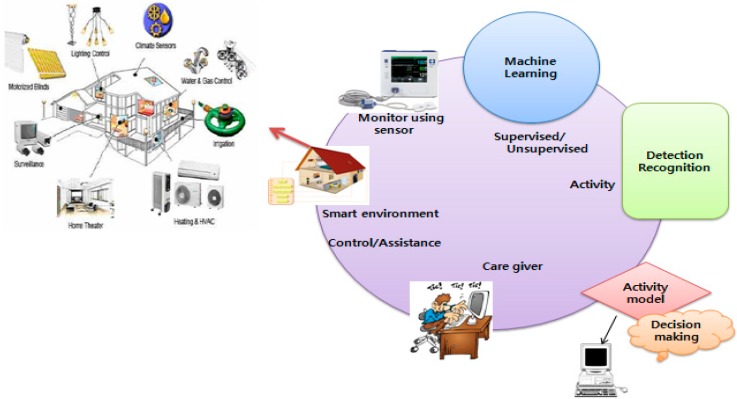
User activity recognition in smart home [1].

Users’ daily activity generate patterns that play an important role in the smart home environment. These patterns are used to favor the recognition of user activity that is useful to improve the smart home applications in terms of efficiency and management energy, healthcare and security as shown in Figure 1. Indeed, the user activities inside the smart home environment have to be monitored and recorded in order to facilitate their control from the remote. Thus, user activity recognition gives the location and time of an activity. According to Figure 1, the abnormal activities in the user behavior can be revealed by constructing the normal behavioral patterns. So, Figure 1 describes the user monitoring in the smart home environment by using object sensors whose collected information is given to the machine learning algorithm as input. In addition, this information is processed by the system to detect anomalies in the user behavior. Therefore, the user can be assisted remotely after receiving an alert message if any unwanted behavior is revealed. Thus, one of the key points of this monitoring system is the ability to provide a response by recognizing the normal user behavior. Furthermore, the following Figure 1 describes the user monitoring in the smart home environment.

The aim of this work is to discuss the possibilities of recognizing and predicting user activities in the smart home environment. If we can develop an accurate activity recognition method, it can be implemented into the smart home control system. Based on the activity recognized by this method, the smart home can provide the appropriate service to the user automatically.

However, the activity recognition is challenging in the real world due to the variability and the complexity of user activities that affect the accuracy of recognition processes. Many researches, which will be introduced in the next chapter, have suggested the ways for the activity recognition, but the accuracy did not reach expectations. The reason is that they focused on only a subpart and not the entire work. Overall, the activity recognition process is made up of two parts. First, the repeated patterns must be discovered and classified from a lot of activities. Second, what action the pattern means should be decided upon. Most previous works dealt with only one of these two points. It is hard to obtain optimal performance if any issue is not investigated from the overall viewpoint.

**Figure 2:**
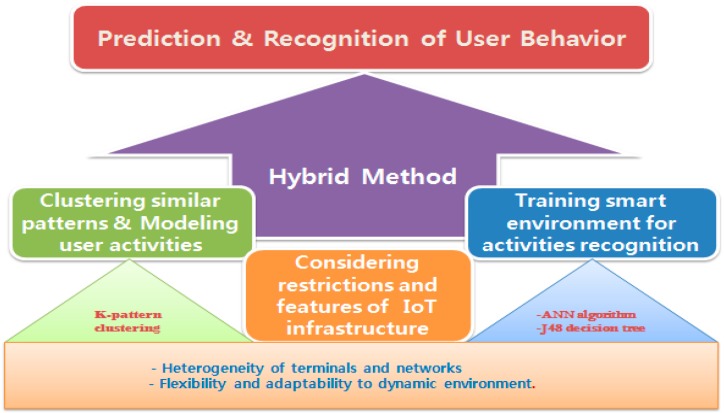
Architecture of hybrid method.

The contribution of this paper comes from this point. Among various existing methods, this paper suggests the best combination of a pattern clustering method and an activity decision algorithm, considering the features of the IoT, especially the smart home environment. As a result, our research takes two steps. First, in order to detect repeated patterns or anomalous user behavior from varied and complex user activities, we investigated existing works extensively. Among them, we chose the K-pattern clustering algorithm [1] because it shows the best performance in terms of the temporal complexity and cluster set flexibility even for the very large amount of data in the IoT smart home environment. The detailed explanation will be given in Section 3.1. On the other hand, the second step describes the training of smart environments for predicting and recognizing user activities inside his/her personal space in order to mitigate the issues related to that activity recognition in the real world. Here, the monitored user plays an essential role in the differentiation of daily activities and habits of each individual. In other words, this step is able to represent the user activities and their variations, and also to recognize those activities when they occur in the smart home environment. Our experimental results in Section 4 showed that the Allen’s temporal relations [2] based artificial neural network (ANN) gives the highest accuracy for user activity recognition. However, this accuracy is achieved at the cost of the run-time as shown in Section 4.2. We suggest the additional use of an efficient feature selection approach called the J48 decision tree to improve both the average accuracy and the run-time performance as shown in Figure 2. This hybrid method deals with the activity recognition challenges, considering the restrictions and features of the IoT based smart home environment.

Most previous researches have showed their limits, especially when they face some challenges such as “concurrent activity recognition”, “discontinuous and interleaved activity recognition”, interpreting different meanings and different time sequences, *etc.* On the contrary, our hybrid method is more accurate and extensible to a dynamic environment such as the smart home. It allows the detection of anomalous or unexpected behaviors, and the mitigation of activity recognition issues in the real world. So, the achievement of this goal is possible by applying the K-pattern clustering algorithm to the temporal based neural network learning algorithm for unsupervised classification of recognizing and predicting user behavior in the smart home environment.

Additionally, unlike conventional detection methods, our hybrid approach considers the problem of heterogeneity and scalability in the smart home infrastructure, focusing on security mechanisms and resident comfort enhancement. 

The remainder of this work is organized as follows: In the next section, the related work is introduced. In Section 3, a theoretical description of the proposed method is presented. Section 4 provides experimental analysis. Finally, the conclusion and future works are offered in Section 5. 

## 2. Related Work 

Various researches have been proposed in the IoT based smart home environment to enhance the security, safety and comfort of residents with minimal operation costs. Obviously, inside smart homes, the use of sensors is indispensable for tracking user activities. Activities daily living (ADL) of users is monitored and the general activity patterns are modeled according to the user position in his/her environment. Thereby, any anomalous or unexpected behavior of the activity pattern can be detected. Moreover, other researches have been employed to mitigate the activity recognition issues with different approaches in various real world activities. However, the diversity and complexity in activities are often very high in daily living.

In [3], the EM-algorithm is used to form groups of similar objects. The algorithm is simple and fast but its efficiency depends on the number of input features, the number of objects, and also the iteration number.

Jakkula *et al.* [4] suggest to partition with a centroid by using the k-means clustering approach. A distance measurement scheme assigns a score to a cluster with the minimum value. However, the efficiency of the algorithm depends upon the number of clusters, the selection of the cluster center, and the number of iterations.

In [5], a hierarchical clustering algorithm is used in a distributed environment to measure its performance and accuracy by applying validation measures like entropy, coefficient of variance and time. The number of clusters needs not to be determined in advance and also easy to be implemented. However, the hierarchical algorithm produces poor quality of clusters and takes a long time for execution when a huge dataset is given. 

The SOM algorithm [6] provides the higher accuracy in classifying objects into their suitable clusters. Moreover, it gives better results compared to the k-means and the EM-clustering algorithm when using random datasets. However, as the number of clusters k increases, the performance decreases considerably; or when using a huge dataset, the SOM algorithm shows poor results.

In general, the existing clustering algorithms above have some ambiguity in processing noisy data. Indeed, this noise makes it difficult to include an object into a certain cluster because it affects the results of the algorithms. In contrast, the K-pattern clustering algorithm has the ability to overcome this drawback.

On the other hand, some works integrate user behavior through activity recognition. Detecting user activities usually implies the collection of observation sequence in order to recognize new events. Some approaches to the activity prediction include sequential activity prediction using the decision trees, the k-nearest neighbor, and the Markov or Bayesian models.

Alam *et al.* [7] use probabilistic models such as Hidden Markov Models (HMM) for modeling user activities. It is a widespread method for identifying the spatio-temporal relationships between the sensor data and also for finding the time series forecasting [8,9]. However, the run-time is very long for huge data volumes. 

In [10], another classification method for activity recognition is considered based on C4.5 classifier. This technique provides good enough results. However, its performance in terms of the recognition accuracy is less than the neural network algorithm due to the diversity and complexity of activities in the real world [11].

In this work, we use the neural network algorithm based temporal relations to overcome these drawbacks related to the recognition accuracy and long run-time, especially with various, complex and large volume of data. 

## 3. Proposed Method

As we mentioned before, the goal of this paper is to suggest the best combination of a pattern clustering method and an activity decision algorithm especially for the smart home application. People behave differently from each other although they do something with the same purpose. For instance, when they prepare breakfast, someone turns on a toaster first while someone opens a refrigerator first. Even one person may show a subtlely different order of behaviors in preparing the breakfast everyday. Therefore, the capability to tell related behaviors apart from a lot of user behaviors is critical as the first step for the activity recognition. To the best of our knowledge, the K-pattern algorithm [1] most efficiently handles so large amount of data in terms of the proximity between elements in the same cluster and the runtime. After the clustering, the ANN method [2] predicts what activity some sequential behaviors mean. ANN shows the best accuracy on the activity prediction although it takes much time. Important features of the K-pattern and ANN methods, which make them the best combinatorial scheme for user activity recognition, are explained in this chapter in detail.

### 3.1. K-Pattern Clustering Algorithm

**Figure 3:**
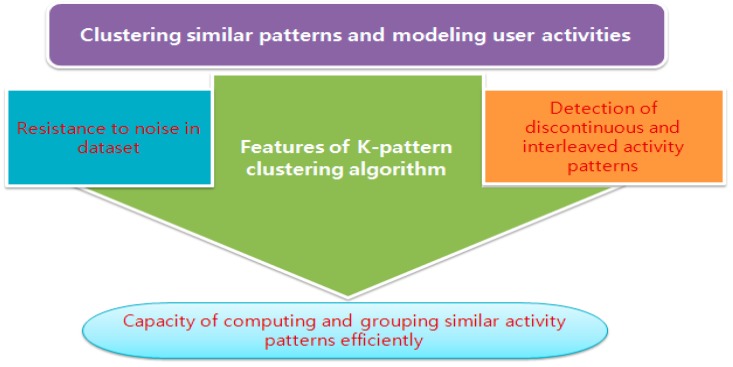
Features of K-pattern algorithm.

In order to train the machine learning algorithms, a huge amount of data is collected from many sensors in the IoT. However, due to the use of this large quantity of data, an unsupervised learning algorithm is preferred to a supervised learning algorithm. Indeed, the clustering algorithm has the capability to efficiently compute data and group similar user activity patterns into clusters. The K-pattern algorithm provides more suitable features than are offered by popular existing partitioning and hierarchical techniques in terms of pattern clustering. Among the major characteristics of the K-pattern algorithm, we underscored the ability to detect discontinuous and interleaved activity pattern of users, the resistance to noise in dataset, and the capability to efficiently compute data and to group similar activity patterns as shown in Figure 3. Noise in datasets makes it difficult for algorithms to group an object into its suitable cluster and affects the results of the algorithm.

**Figure 4:**
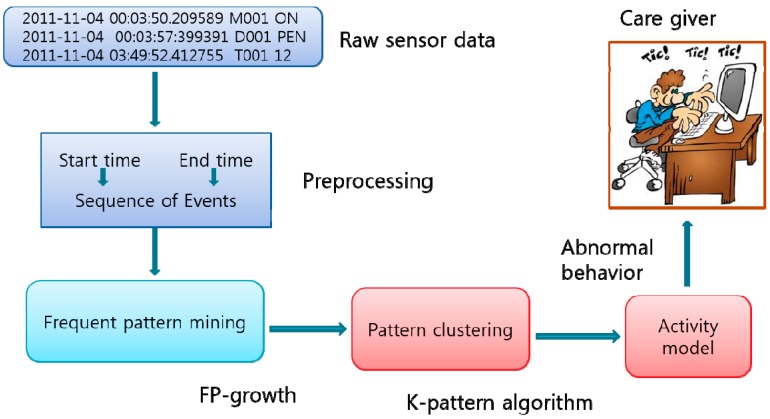
Architecture of tracking activity using K-pattern algorithm [1].

The use of the K-pattern clustering algorithm should lead to the detection of temporal relations by processing perceived data. Indeed, the processing of perceived data constitutes the first step in activity recognition of users in the smart home as seen in Section 5. Thus, the pattern clustering algorithm complies with a methodology consisting of the perceived sensor data conversion stage, the most frequent pattern observation and mining stage, and the similar pattern grouping stage. The perception data are converted to a symbolic representation in order to facilitate the similarity between collected data and current normal pattern into sequences of events as illustrated in Figure 4. Among popular existing techniques, the symbolic aggregation approximation (SAX) algorithm [12] is adopted. The outstanding advantage in terms of requiring imperceptible time and space overhead makes SAX most efficient in the IoT based smart home environment. The perception data collected within time interval *t* is defined as a time series as: {S*1*, S*2*… S*t*}. By using SAX, data can be defined as a string of alphabet as:{C*1*, C*2*… C*n*}, where *n* denotes the length of the symbolized string, should be much less than *t*. The converted sensor data makes up of date and time when the data was collected, sensor identification (ID), and the state of the corresponding sensors (ON/OFF) or (OPEN/CLOSE). This step is defined as preprocessing of perceived data. The sensor data can be labeled, for example, by considering sensor motion represented by “M”, sensor temperature by “T” and sensor door by “D” as shown in Table 1. This data came from the Washington State University (WSU) CASAS smart home project [1,13]. They include a variety of user behaviors and the related user activities which emulate the training operation for the ANN method.

**Table 1:** Sensor data representation [1].

Date	Time	Sensor ID	Sensor State
4 November 2011	00:03:50 209589	**M001**	**ON**
4 November 2011	00:03:57 399391	**D001**	**OPEN**
4 November 2011	03:49:52 412755	**T001**	**12**

The detection of frequent patterns is noticed when their frequency of occurrence in the dataset is greater than or equal to a specific threshold. Further, these frequent patterns are extracted using some frequent pattern mining algorithms such as the frequent pattern growth (FP-growth). Indeed, the FP-growth proposed by Han *et al.* [14] is an efficient and scalable technique for extracting the entire set of frequent patterns. This method uses an extended prefix-tree structure to store compressed and crucial information about frequent patterns [15,16]. According to [17,18,19], it was demonstrated that the FP-growth is as successful as other techniques, such as the Eclat method for fast discovery of association rules [20], a recursive elimination method, Relim, to find frequent item sets [21] and the Apriori algorithm [15]. Therefore, the FP-growth is given as input to the K-pattern clustering and its efficiency contributes widely to the identification of frequent activity patterns of user behavior in the smart home environment. The last stage is to group similar patterns by using the frequent activity patterns’ mining.

**Figure 5:**
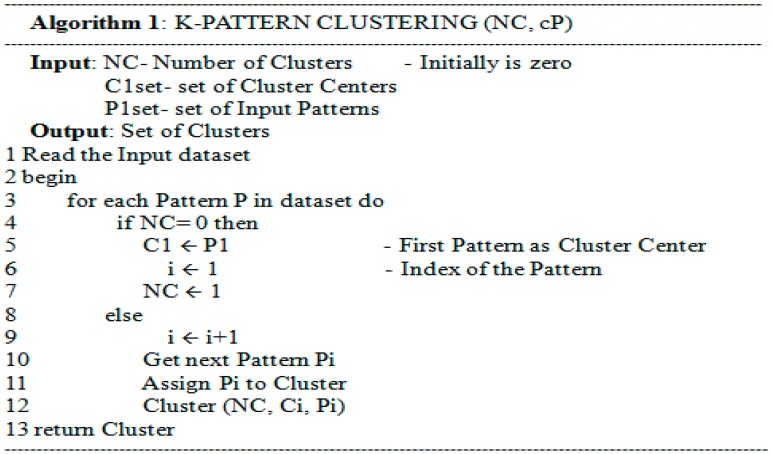
Process of forming frequent activity patterns [1].

The pseudo code for methodology analysis is described in the following Algorithms 1–3. The set of frequent activity patterns and the number of clusters are the input, while the set of clusters is the output of the algorithm as shown in Figure 5.

**Figure 6:**
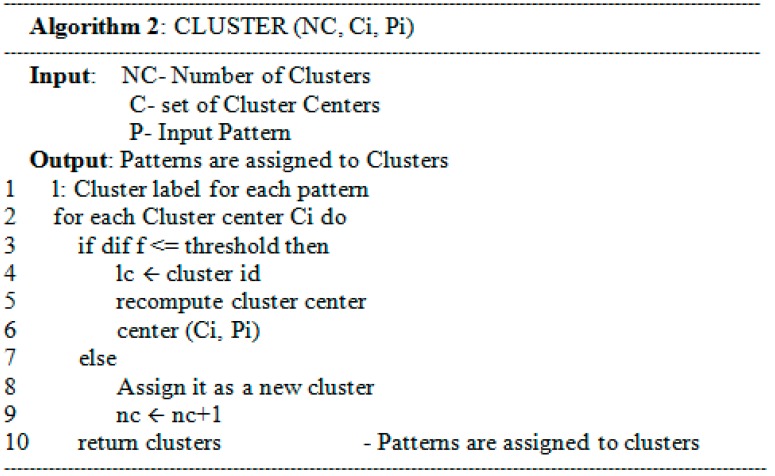
Process of forming clustering [1].

**Figure 7:**
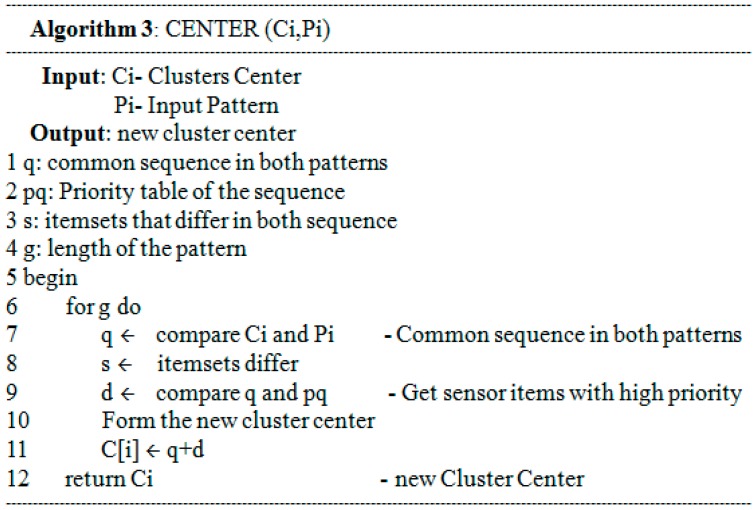
Process of recomputing new center [1].

Two patterns belong to the same cluster if the distance between them is less than a specified threshold. In that case, a new cluster center has to be computed as Lines 3–6 in Figure 6. In Figure 7, the computation of a new cluster center firstly needs to compare the sequence length to get the common items from both the input pattern and the cluster center (Line 7). The second stage gets different items from this input pattern and the cluster center to check the priority table in order to get the sequence with the highest priority (Lines 8–9). Finally, the items formed at Lines 8 and 9 are combined to create a new cluster center (Line 11). 

#### 3.1.1. Performance Evaluation of K-Pattern Algorithm

**Figure 8:**
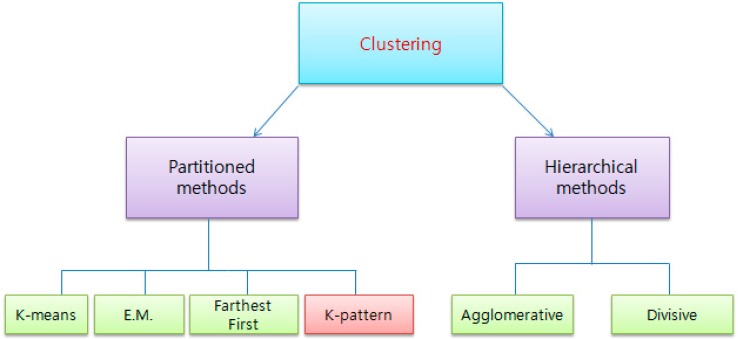
Basic taxonomy of clustering algorithm.

In the following, we realize a comparative study between the K-pattern clustering algorithm and some partitioned algorithms (K-means clustering, expectation maximization algorithm, Farthest First) and hierarchical algorithm. Refer to Figure 8 for the basic taxonomy of the clustering algorithms.

The comparison of these clustering algorithms considers the parameters related to dataset size, the number of clusters, and running time to form clusters. To achieve this task, we use WEKA 3.7.11, a popular open source machine learning software package implementing many state of the art machine learning algorithms. It contains tools for data pre-processing, classification, regression, clustering, association rules and visualization and consists of four interfaces as follows: Explorer, Experimenter, Knowledge Flow, and Simple CLI. These useful intefarces are the main reason why WEKA is widely used.

Using WEKA, we compared the running time and the resulting number of clusters of various clustering algorithms. In order to see the effect of dataset size, we downloaded datasets with various sizes from WEKA official web [22]. The numbers of attributes and instances of each dataset are given in Table 2.

**Table 2:** Dataset collected for running time.

Dataset Name	Number of Attributes	Number of Instances
**Dataset 1**	5	150
**Dataset 2**	9	1253
**Dataset 3**	9	2924

Table 3 summarizes the running time to form clusters for three different sizes of datasets. In general, when the size of dataset increases, the running time to form clusters also increases. The K-pattern algorithm and Farthest First take the least time to form clusters for all three cases of datasets, while Expectation Maximization (EM) and the hierarchical algorithm take the longest time.

**Table 3:** Running time for cluster forming.

Algorithm	Running Time with Dataset 1	Running Time with Dataset 2	Running Time with Dataset 3
**Hierarchical**	0.19	5.52	28.15
**EM**	1.96	121.98	963.40
**K-means**	0.02	0.04	0.18
**Farthest First**	0.01	0.03	0.1
**K-pattern**	0.01	0.02	0.09

Table 4 describes the resulting number of clusters formed by each clustering algorithm for different sizes of datasets. Since the default value for the number of clusters is given in WEKA, most clustering algorithms form the same number of clusters regardless of the size of datasets. However, the K-pattern clustering and EM algorithm form the more clusters when the size of the dataset increases. The K-pattern clustering algorithm form the largest number of clusters, which means the data patterns are classified more specifically.

**Table 4:** Number of clusters formed using different datasets.

Algorithm	Number of Clusters with Dataset 1	Number of Clusters with Dataset 2	Number of Clusters with Dataset 3
**Hierarchical**	4	4	4
**EM**	7	12	21
**K-means**	4	4	4
**Farthest First**	4	4	4
**K-pattern**	9	14	25

#### 3.1.2. Interpretation of Results

In our work, using three different datasets, we observed the running time to form clusters is roughly proportional to the data size. The results showed that K-pattern clustering algorithm took the least time in forming clusters, and the EM algorithm took the most time. Meanwhile, some clustering algorithms such as Farthest First, K-means, and Hierarchical always formed the same number of clusters irrespective of dataset sizes, while the K-pattern algorithm formed an increasing number of clusters as the dataset size grows. Indeed, K-pattern formed the most clusters when the data size was large. 

Conclusively, our comparative study showed that the K-pattern clustering algorithm provides the best performance in terms of running time and the number of clusters. This good performance comes from the capability of the K-pattern algorithm to efficiently compute data, to resist noise in dataset, and to detect discontinuous and interleaved user activity behavior. 

### 3.2. Artificial Neural Network Algorithm 

**Figure 9:**
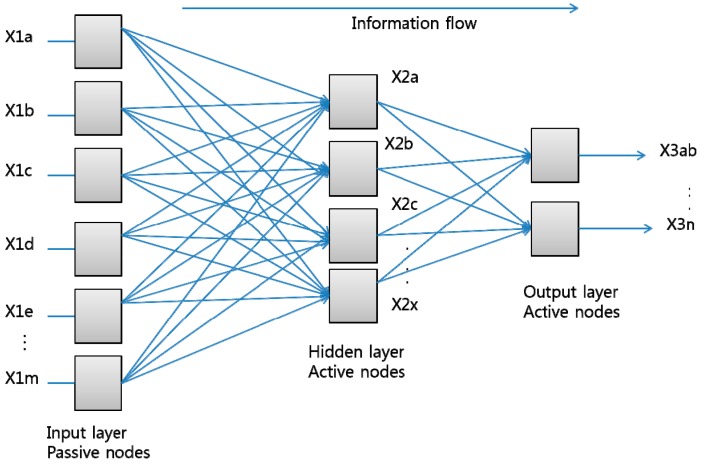
Multilayered artificial neural network.

Artificial Neural Network (ANN) is used to make a system learn a mapping for attribute values that can be applied to classify new and hidden anomalous behaviors. Being inspired by the architecture of the human brain, ANN is composed of inter-connected nodes and weighted links. Nodes in an ANN are called neurons as an analogy with biological neurons [8]. Various network architectures have been designed depending on actual activity fields. The most widely used one is made up of three layers called the input layer, hidden layer, and output layer, where each of them consists of one or more nodes represented by the rectangles in Figure 9. The information flow is symbolized by the lines between one node and the next one. The nodes in the input layer receive a single value on their input and duplicate the value to the multiple outputs without modifying data. Meanwhile, the nodes in the hidden and output layers actively modify the data. These simple functional units are composed of networks that have the ability to learn a classification problem after they are trained with sufficient data. The ANN is suitable for user activity recognition and prediction. In order to recognize user activities, the Multilayer-Perceptron learning method is chosen. Figure 9 illustrates its common architecture.

**Figure 10:**
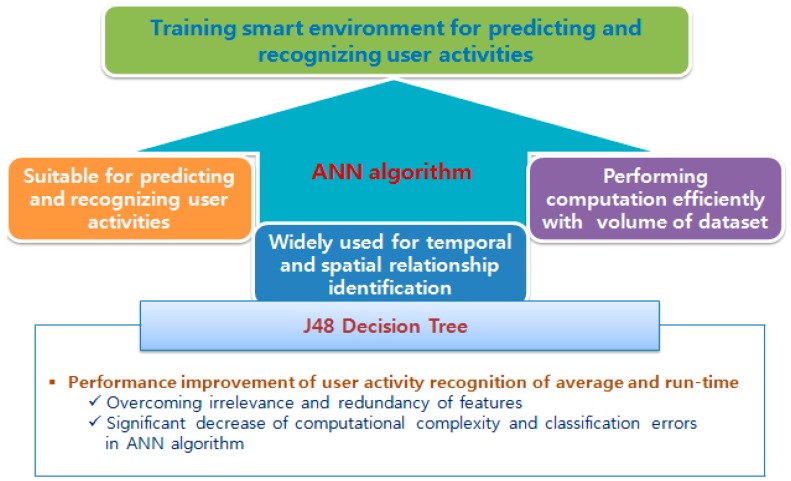
Training smart environment for activities recognition.

The ANN algorithm is characterized by its capability to efficiently recognize and predict user activity, to perform computation efficiently despite the large volume of dataset, whereas other classification techniques such as Hidden Markov Model (HMM), Naive Bayes, and C4.5 face challenges in terms of the runtime and interleaving events. On the other hand, the ANN algorithm is widely used for temporal and spatial relationship identification. Thus, as we said previously, besides the ANN algorithm, the J48 decision tree is added in order to overcome the irrelevance and redundancy of features that can significantly increase the computational complexity and classification errors of the algorithms, especially ANN. Figure 10 illustrates the training for a smart home system to recognize and predict user activities using ANN based on the Allen’s temporal relations.

### 3.3. Temporal Relations

In the IoT based smart home environment, the sequences of events also called activities are mainly physical as well as interactive with objects. For example, after playing a sport, he/she uses a mobile phone while taking a rest. These activities are temporarily indicated by timestamp in the datasets. Here, we note that the start and the end time of any activity should not be too close to each other. Otherwise, temporal relations between several events belonging to an activity can be switched unexpectedly. 

**Figure 11:**
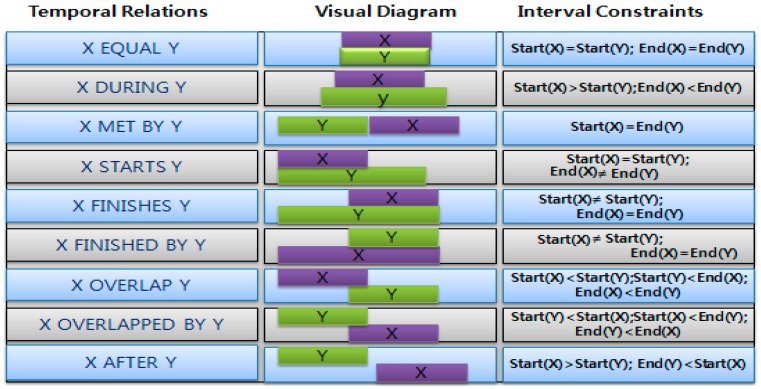
Temporal relations representation.

For any normal activity, a temporal link can be made between sequential events. These relationships can be represented using the Allen’s temporal relations for pattern discovery in user’s daily activities. On the other hand, temporal relations are necessary in either anomaly detection or anomaly alert. In [8], activity scenarios in a set of temporal relations are illustrated using the Allen’s interval algebra like Figure 11.

## 4. Experimental Analysis

In this section, the possibility of implementing the user activity recognition inside the smart home is discussed. First of all, our analysis aims to find the most important temporal relations by assessing some classification algorithms. To achieve this goal, we consider a set of events from each activity, which have the temporal relations between them. These temporal relations are used to recognize activities that have been undertaken. Furthermore, a sequence of these temporal relations between successive events defines the type of activity in the dataset. These temporal relations of successive event are detected by some algorithms such as FP-growth [15,16] and Apriori algorithm [17], based on the importance degree of each activity. The importance degree is the sum of the probability that each sequence of events can be discovered when any activity detected in a dataset is input to a pattern algorithm. This characterizes each activity and always impacts the activity recognition. Consequently, if a specific sequence of events is discovered, the recognition method determines that it belongs to the activity with the highest importance degree.

Meanwhile, for the accurate analysis, a temporal relation must occur for at least the minimum number, then this minimum number is dynamically changed in this work depending on the total number of events in each activity. If the minimum number is absolute, any activity including events less than the absolute value cannot be recognized however large a fraction is the most important temporal relation that takes among the total events. Thus, a relative threshold on the minimum occurrence of events for classification algorithms is used considering the number of events during each activity.

The actions we had interest in include taking a bath, preparing breakfast, listening to music, preparing lunch and playing a game. Those actions can be recognized through a set of temporally related activities as shown in Figure 11. For example, if a light in the bathroom is turned on, the faucet in the bathtub is turned on and left running for some time, and the light is turned off more than 15~20 min later, then we can guess an occupant took a bath. On the other hand, if a motion sensor in the dining room detects a person, the refrigerator door is opened, and the heat increases around an oven, then we can think a resident is preparing a meal. The meal at 11:00 a.m. may be thought as either breakfast or lunch depending on his or her daily routines that have been observed for a long time. Some sound or acoustic sensors can be used to know whether a dweller is listening to music or playing a game.

The data related to those activities were taken from [23] and simulated for different periods, *i.e.*, for two weeks and one week, respectively. A total of 77 and 84 sensors were attached to electric devices and appliances in two apartments of a 30-year old and a 80-year old woman, respectively, and the on and off information of corresponding devices was collected for 14 days. Using different methods such as the neural network algorithm, C4.5 classifier, naïve Bayes, and HMM, we evaluated the activity recognition accuracy for the most important activities as well as the runtime performance. As aforementioned, WEKA provides an implementation of the learning algorithms that are easily applied to our dataset [24]. With WEKA, the selected learning classification algorithms were evaluated by using 10-fold cross validation.

Further, in order to reduce classification errors and computational complexity caused by redundancy and irrelevance of data, we adopted the feature selection method of J48 decision tree [25]. Indeed, this is one of the most popular feature selection methods to improve the performance in terms of running time and accuracy. The J48 decision tree feature selection method utilizes a sequence of attributes which has the highest similarity between them in a given set of training feature vectors to create a classification model, *i.e.*, a statistical property that measures how useful a given attribute is to separate the training examples according to their target classification [26]. By using the J48 decision tree algorithm, we can learn which feature plays an important role towards the target classification and select several ones as the main features for other machine learning techniques. After all, we compare the running time of ANN, HMM and NB algorithms after making use of a feature selection method. The experimental results substantiated the superiority of the neural network algorithm compared to C4.5 classifier, naive Bayes, and HMM algorithm in terms of average accuracy. 

### 4.1. Comparison of the Recognition Accuracy 

At first, all activities are discovered based on all available data in the common dataset. Then, the activities that most frequently appear are considered as the dominant activities in the smart home environment. Table 5 describes the recognition accuracy by each classifier algorithm for the dominant activities during the period of two weeks. The accuracy is defined as the ratio of successful activity recognitions to the total occurrences of the activity.

**Table 5:** Activity recognition with data of two weeks.

Activity	ANN	HMM	NB	C4.5
**Taking bath**	0.93	0.85	0.85	0.85
**Preparing breakfast**	0.94	0.88	0.88	0.89
**Listening to music**	0.57	0.54	0.52	0.52
**Playing game**	0.94	0.90	0.89	0.91
**Preparing lunch**	0.55	0.53	0.52	0.53

As for the results of two weeks’ training, the method is satisfactory overall, even though the accuracy barely exceeds 50% for certain activities. This low accuracy can be explained by the fact that, these activities include some actions (events) that occur also in different activities. For example, listening to music can be executed simultaneously when a user is opening the faucet in the bathroom or selecting the favorite games on his/her computer. Even though she/he may listen to music while they are either taking a bath or playing a game, the event can be still regarded as listening to music as the major action with high certainty. Therefore, this situation constitutes one of the main difficulties of our dataset and an efficient solution should be found to overcome this issue. On the other hand, the low accuracy for a certain activity may be due to its lower importance than other similar activities. For instance, both preparing lunch and preparing breakfast activities may include some common actions. However, since people more likely eat breakfast at home than lunch, those common actions are considered preparing breakfast as the main activity with higher certainty.

Next, the recognition accuracy of the system was evaluated with the data of one week and the results are given in Table 6. Overall, the results are better than the case with two week data. We guess this is because it is easier to recognize several dominant activities in small datasets. As the number of activity types increases in proportion to the amount of data, there is a higher chance that an activity similar to one of the dominant ones can be included in the two week dataset. The pattern recoginition scheme may confuse the dominant activity and the similar one occasionally.

**Table 6:** Activity recognition with data of one week.

Activity	ANN	HMM	NB	C4.5
**Taking a bath**	0.98	0.92	0.92	0.93
**Preparing breakfast**	0.69	0.66	0.64	0.66
**Listening to music**	0.91	0.87	0.86	0.88
**Playing a game**	0.65	0.61	0.61	0.63
**Preparing lunch**	0.73	0.69	0.70	0.71

However, compared to Table 5, we notice the inversion in the accuracy between listening to music and playing a game. The similar situation is observed between preparing breakfast and preparing lunch. The recognition accuracy for playing a game and preparing breakfast was reduced to around 62.5% and 66.25%, respectively, while listening to music and preparing lunch were recognized more accurately with around 88% and 70.7% accuracy. Indeed, this is due to the similarity between activities within the dataset. For instance, listening to music and playing a game are related activities. Likewise, preparing breakfast and preparing lunch are related. Since the related activities contain the same major actions (events), the distinction between them is very difficult to be made.

Throughout this experiment, we noticed that the increased recognition error for one activity is complemented by the decreased error for another related activity. This tradeoff is controlled by the choices of the most important temporal relations between events. Thus, if closely related activities are merged into one activity, the recoginition accuracy can be significantly improved. For example, by combining preparing breakfast and preparing lunch into a single activity, preparing a meal, we can improve the recognition accuracy. The same can be done by combining listening to music and playing a game into a single activity, entertainment. 

Table 5 and Table 6 show that most algorithms in our study have a good performance in recognizing user activities. The ANN algorithm yields the highest accuracy of about 79% for both datasets, while NB yields the lowest accuracy of around 74%. This ANN algorithm efficiency is due to its interconnecting artificial neurons which provide a general and robust method to learn a target function from input examples. Moreover, since the ANN algorithm is also applied to problems with dynamic or non-linear relationships, it can capture many kinds of relationships that may be difficult to be modeled by other classification techniques. As seen in Figure 9, the Multilayer-Perception learning method is chosen to recognize activities.

Finally, the J48 decision tree feature selection method was added to improve the average accuracy of all algorithms. Table 7 shows that the J48 decision tree can improve the average accuracy of all the algorithms. Especially for ANN, on average, an improvement of 7% can be made. 

**Table 7:** Comparison of average accuracy.

	Comparison of Average Accuracy			
Algorithms	1 Week Dataset		2 Weeks Dataset	
	wo/J48	w/J48 Tree	wo/J48	w/J48 Tree
**ANN**	0.79	0.88	0.78	0.83
**NB**	0.74	0.77	0.73	0.79
**HMM**	0.75	0.82	0.74	0.78

### 4.2. Comparison of the Runtime

The runtime was evaluated for two purposes: First, the comparison between the three algorithms, ANN, NB, and HMM; Second, to see how much the runtime can be reduced by using the J48 decision tree feature selection method. Table 8 gives the runtime of each algorithm in detail without and with the feature selection. The feature selection method was helpful to reduce the runtime for all the algorithms in common. Particularly, the runtime of ANN was reduced to just about 8% as compared to the result without the feature selection method. Despite the improvement by the J48 decision tree, the runtime of ANN is still longer than the other algorithms. However, we argue that this can be complemented by the better performance in terms of the activity recognition accuracy.

**Table 8:** Comparison of runtime.

	Runtime (s)			
Algorithms	1 Week Dataset		2 Weeks Dataset	
	wo/J48	w/J48 tree	wo/J48	w/J48 tree
**ANN**	50.12	3.99	316.80	25.15
**NB**	0.31	0.32	0.10	0.03
**HMM**	0.35	0.56	1.14	0.89

## 5. Conclusions and Future Work

In this paper, we discussed the possibility of recognizing and predicting user activities in IoT based smart environments. Due to the complexity and variety of user activities, we proposed a hybrid approach consisting of the K-pattern clustering and neural network algorithm based on temporal relations. The K-pattern clustering demonstrated its efficiency to group and identify the user activity model. Additionally, the K-pattern clustering is more suitable than others for detecting a discontinuous and interleaved activity pattern.

In the mean time, we suggested an approach for user activity recognition and prediction based on the artificial neural networks, which provided good results in general. However, we also need to resolve some unsatisfactory results caused by the similarity between related activities. The recent adoption of a feature selection approach based on the J48 decision tree significantly improved the recognition accuracy and runtime performance. 

After all, our hybrid method of K-pattern and ANN is more accurate, extensible, and adaptable in a dynamic environment such as an IoT network and is useful for smart home applications. In our future research, we will improve the activity recognition accuracy with the presence of more sensitive sensors to collect more useful information in the smart home environment. Moreover, the application of more efficient feature selection approaches to a classification method to overcome redundancy and irrelevant attributes is desirable.
